# *Bacillus marcorestinctum* sp. nov., a Novel Soil Acylhomoserine Lactone Quorum-Sensing Signal Quenching Bacterium

**DOI:** 10.3390/ijms11020507

**Published:** 2010-02-03

**Authors:** Yan Han, Fang Chen, Nuo Li, Bo Zhu, Xianzhen Li

**Affiliations:** Department of Bio & Food Engineering, Dalian College of Light Industry, Dalian 116034, China; E-Mails: cindy415han@hotmail.com (Y.H.); fchen2007@126.com (F.C.); lijiaxiaonuo@163.com (N.L.); Zhubo_1988@163.com (B.Z.)

**Keywords:** *Bacillus marcorestinctum*, quorum sensing, quench, autoinducer, isolation

## Abstract

A Gram-positive, facultatively anaerobic, endospore-forming and rod-shaped bacterium was isolated from soil samples and designated strain LQQ. This organism strongly quenches the acylhomoserine lactone quorum-sensing signal. The LQQ strain exhibits phenotypic characteristics consistent with its classification in the genus *Bacillus*. It is positive in catalase and no special growth factor is needed. It uses glucose as sole carbon source. The DNA G *+* C content is 39.8 mol %. The closest relatives based on the 16S rRNA gene sequence are *Bacillus anthracis*, *Bacillus thuringiensis*, and *Brevibacillus brevis* (syn. *Bacillus brevis*) with the similarity of 96.5%. The DNA–DNA hybridization data indicates a low level of genomic relatedness with the relative type strains of *Bacillus thuringiensis* (6.1%), *Bacillus anthracis* (10.5%) and *Brevibacillus brevis* (8.7%). On the basis of the phenotypic and phylogenetic data together with the genomic distinctiveness, the LQQ strain represents a novel species of the genus *Bacillus*, for which the name *Bacillus marcorestinctum* sp. nov. is proposed. The type strain is LQQ^T^.

## 1. Introduction

The term “quorum sensing (QS)” has been proposed to describe a cell-cell communication mechanism used by microbes to monitor their own population density and synchronize the expression of virulence genes to thus mount effective attacks and overwhelm a host’s defense responses [1]. QS is now recognized as playing a major role in the virulence of pathogens [2]. It has been suggested that targeting the QS system should allow the development of valuable biological control agents with the advantage of less powerful selection towards resistance [3]. The signal molecule autoinducer such as acylhomoserine lactone (AHL) is the key factor in the cell-cell communication process, which controls the virulence factor production such as plant cell wall degrading enzymes cellulase, protease, and pectinase. Degradation of the autoinducer can prevent its sufficient accumulation in the immediate vicinity of the bacterial cells and consequently leads to an attenuation of the QS response. This has been verified in the simple co-culture of the AHL-degrading bacterium and AHL-producing bacterium [4].

The approaches disrupting bacterial QS by degradation of AHL autoinducer, termed as quorum quenching, have attracted a great deal of interest because of their potential therapeutic applications. Such metabolic inactivation activity in some bacteria is due to its synthesis and secretion of autoinducer-digesting enzymes. To date, three kinds of enzymes including lactonase, acylase and oxidoreductase have been found to be capable of quenching QS [5–7]. AHL lactonase hydrolyses the homoserine lactone ring of molecule AHLs to open the homoserine lactone ring, thereby reducing the effectiveness of the signal molecules [8]. AHL acylase hydrolyses the amide linkage between the acyl chain and the homoserine moiety of AHL molecules releasing homoserine lactone and the corresponding fatty acid, which does not exhibit any residual signaling activity [9]. Oxidoreductase converts 3-oxo-AHLs to their corresponding 3-hydroxy derivatives that may be degraded by amidohydrolase to form homoserine lactone and hydroxydecanoic acid [10].

Over the last decade, researchers have documented a diversity of microbes capable of rapidly degrading AHL autoinducers. The first report on such degradation was the isolation of *Bacillus* sp. 240B, in which AHL lactonase was cloned and expressed in transformed *Pectobacterium carotovorum* significantly reducing the release of autoinducer [8]. Subsequently, database searches for homologues of the lactonases and acylases in complete bacterial genomes have shown the existence of the related enzymes in a wide range of species. However, most of the characterized microbes are spread among the QS-mediated pathogens [5,6,11], and only a few data related to AHL degradation are from non-QS bacteria [8,9,12,13]. Much effort is now being invested in searching the potential quorum quenchers and their roles in autoinducer-mediated mechanisms in QS pathogen, there is a little attention being paid to the autoinducer-depleting microbes.

In searching for novel quorum-quenching bacteria from soil samples, we obtained an isolate that strongly inactivated autoinducing activity and reduced the severity of plant soft-rot, which could potentially be a biological control for plant soft rot. In this paper, a description of this novel autoinducer-quenching strain was presented, including its cultural and biochemical characteristics, as well as its phylogenetic position based on 16S rRNA gene sequence information. We have concluded that this strain should be classified as a new species of the genus *Bacillus*, and the name *Bacillus marcorestinctum* sp. nov. is proposed for this strain, with type strain LQQ^T^.

## 2. Results and Discussion

### 2.1. Isolation of the Autoinducer-Quenching Strain

After incubation of the mixture of the culture supernatant and the synthetic autoinducer for 3 h and determining its residual autoinducing activity, fifteen isolates capable of completely quenching autoinducer activity were obtained from about 650 colonies screened. When the incubation time of the culture supernatant with the synthetic autoinducer was shortened to 1 h, only four isolates were found to be able to quench autoinducer activity completely. One single colony, which showed a strong ability to eliminate autoinductive activity, was selected from those four colonies as the target strain by cut down incubation time further. The isolate was purified further on YEB agar plates by streaking and the purified species was designed as LQQ^T^ (T = type strain).

**Figure 1.**
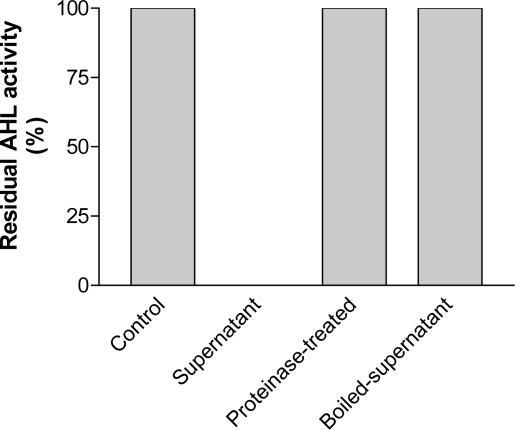
The residual AHL activity after the synthetic autoinducer was incubated with the culture supernatant, the proteinase-treated supernatant, or the boiled supernatant of the LQQ strain, in which the untreated synthetic autoinducer was used as control test and its AHL activity was expressed as 100%.

As shown in Figure 1, the culture supernatant of the isolate LQQ can eliminate autoinducer activity completely in 30-minute incubation with the synthetic autoinducer. However, the autoinducer-quenching activity was abolished completely when the culture supernatant of LQQ strain was treated with proteinase for 2 h or heated in the boiling water for 30 min before incubation with the synthetic autoinducer.

### 2.2. Severity Reduction of Potato Tuber Soft-Rot

**Figure 2.**
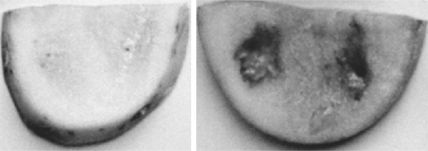
Potato inoculation with *Pectobacterium carotovorum* (right) and *Pectobacterium carotovorum* together with LQQ strain (left).

To assess whether strain LQQ co-incubated with pathogen *P. carotovorum* reduces the severity of soft-rotting of potato tubers, the potato tuber assay was performed and a pathogenicity assay with three replicates is shown in Figure 2. When the inoculum of the LQQ isolate was applied to sliced potato tubers together with *P. carotovorum*, the soft rot symptoms caused by *P. carotovorum* could be effectively attenuated. It can be concluded that the LQQ isolate was able to inactivate the AHL autoinducers produced by the rotting bacterium *P. carotovorum*, leading to the reduction in the severity of potato decay.

### 2.3. Colony and Cell Morphology

The isolate LQQ is an endospore forming but not a flagellated bacterium. Its colonies are circular, flat and erose, rough and opaque, and the texture is white and dry. The cells from young cultures of LQQ strain were straight rods with round ends, usually 0.7–1.0 μm in diameter and 3.0–4.6 μm in length. They occurred singly and sometimes in chains.

### 2.4. Physiological and Biochemical Characteristics

The physiological and biochemical properties are shown in Table 1. The LQQ strain was a Gram-positive and facultatively anaerobic bacterium. The isolate was positive in catalase, urease, nitrate reductase and egg-yolk lecithinase, but negative in oxidase and lipase. LQQ digested gelatine, starch and casein, but not cellulose. Hydrogen sulphide can be produced from cystein but not from triosulfate and TSI. The LQQ isolate can use glucose as a sole carbon source but not utilize citrate. Acid was produced from the carbon sources including glucose, trehalose, maltose and glycerol, but never from the carbon sources sucrose, lactose, fructose, manitol, inositol, mannose, galactose, ethanol and sorbitol. All the tested carbon sources above supported the cell growth of LQQ strain, in which no gas was detected. The nitrogen sources such as peptone, tryptone, yeast extract, beef extract, casein hydrolysate and (NH_4_)_2_SO_4_ supported the growth of LQQ isolate, whereas urea and KNO_3_ did not. The autoinducer-quenching activity can be detected in the basal medium with all the tested carbon sources. The isolate grew at temperatures ranging from 10 °C to 37 °C with an optimal temperature at 30 °C. No special growth factors were needed when the LQQ isolate was grown in a medium with glucose as sole carbon source.

### 2.5. Phylogenetic Analysis

To establish the phylogenetic position of the isolate LQQ, 16S rRNA gene from the isolate LQQ was sequenced and a 1275-base sequence was obtained (accession GQ900516, GenBank). Preliminary comparative sequence search in the EMBL/GenBank libraries and Ribosomal Database Project-II reveled that 16S rRNA gene sequence of strain LQQ was most similar to that of the species belonging to the genus *Bacillus* [14,15].

**Figure 3.**
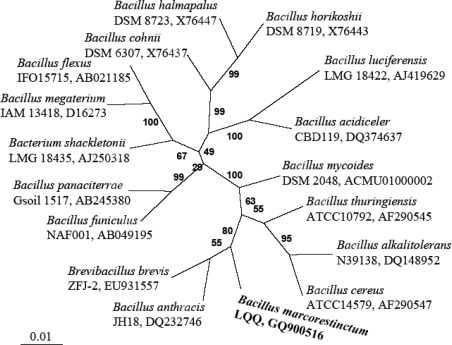
Neighbor-joining phylogenetic tree showing the position of the isolate LQQ^T^ within the genus *Bacillus* based on 16S rRNA gene sequence data. GenBank accession numbers were shown in parentheses. Bootstrap values expressed as a percentage of 100 replications were given at the branching points. The scale bar represents 1% sequence dissimilarity.

The similarity matrix derived from the sequences being most similar to strain LQQ 16S rRNA gene sequence were calculated with Megalign program in DNAStar software package and shown in Table 2. The closest relatives of strain LQQ were the type species of *B. anthracis*, *B. thuringiensis*, and *Brevibacillus brevis* (synonym *Bacillus brevis*), in which the 16S rRNA gene sequence similarity of LQQ strain towards all three strains was 96.5%. LQQ strain also showed high sequence similarities with other phylogenetic neighbors, namely *B. cereus* (96.4%), *B. mycoides* (96.2%) and *B. alkalitolerans* (94.6%), respectively. The data set used for the construction of the phylogenetic tree contained 1,275 base pairs of each sequence as a result of elimination of gaps and ambiguous nucleotides from the 16S rRNA gene sequences between positions 95 and 1395 (*Escherichia coli* position numbers). The phylogenetic tree constructed by the neighbor-joining method is shown in Figure 3. Our strains formed a phylogenetic cluster with *B. anthracis* and *Bre. brevis*.

### 2.6. DNA Base Composition and DNA-DNA Hybridization

Based on the T_m_ data determined *via* thermal denaturation, the guanine-plus-cytosine (G + C) content of LQQ strain was calculated to be 39.8 mol %, which is comparable to that of the genus *Bacillus,* ranging from 32% to 69%. The levels of DNA-DNA association between the LQQ isolate and the related *Bacillus* specie were determined. The LQQ isolate exhibited very low hybridization levels with *B. mycoides*, *B. cereus*, *B. thuringiensis*, *B. anthracis* and *Bre. brevis* of 4.3%, 4.8%, 6.1%, 10.5% and 8.7%, respectively.

### 2.7. Discussion

Quorum quenchers have been documented in a diversity of microbes, most of which are plant- and soil- associated strains, distributed among the QS-dependent pathogens and saprophytic microbes [5]. The novel LQQ strain showed the capability of quenching autoinducer activity. As shown in Table 1, the taxonomic properties of the LQQ isolate were consistent with the key characteristics of the genus *Bacillus* including rod-shaped cells, endospore formation, Gram-positive, facultatively anaerobic and catalase formation [16]. Therefore, LQQ strain should be phenotypically classified in the genus *Bacillus*.

Phylogenetic analysis based on the 16S rRNA gene sequence also supported the assignment of the newly isolated bacterium to the genus *Bacillus*. As shown in the neighbor-joining phylogenetic tree (Figure 3), the LQQ isolate was clearly clustered into the clade of the genus *Bacillus*, in which the closest species were *B. anthracis*, *B. thuringiensis*, and *Bre. brevis* with the similarity of 96.5% and *B. cereus*, *B. mycoides* with similarities of 96.4% and 96.2%, respectively. These data were in accordance with the proposal of 95% 16S rRNA gene sequence similarity as a cut-off value for delineating genera [17].

DNA–DNA hybridization data indicated that the LQQ isolate was distinct from *B. mycoides* (4.3%), *B. cereus* (4.8%), *B. thuringiensis* (6.1%), *B. anthracis* (10.5%) and *Bre. brevis* (8.7%). These hybridization values were sufficiently lower than the recommended threshold value accepted for defining a novel species [18,19]. Therefore, based on the more than 3% difference at the 16S rRNA gene sequence level and less than 70% similarity at the DNA–DNA relatedness level with the closest related *Bacillus* species, LQQ strain can be classified as representing a novel species within the genus *Bacillus*. This classification also is supported by several phenotypic differences of the LQQ strain with other phylogenetic neighbors (Table 3).

On the basis of the differential phenotypic and phylogenetic characteristics, as well as DNA-DNA hybridization data, it is apparent that the LQQ strain cannot be assigned to any previously recognized bacterial species. We propose therefore the creation of a new species within the genus *Bacillus*, to be named *Bacillus marcorestinctum* sp. nov. A description of the type strain is given below.

### 2.8. Description of Bacillus marcorestinctum sp. nov.

*Bacillus marcorestinctum* (mar.co.res.’tinc.tum. L. n. marcor, rottenness; L. adj. restinctio, quenching; N. L. neut. adj. marcorestinctum, rottenness-quenching, intended to reflect ability to quench autoinducer activity and also plant decay). Gram-positive, facultatively anaerobic, rod-shaped organism occurring singly and sometimes in chains. Cells range from 0.7–1.0 μm in diameter to 3.0–4.6 μm in length. They form oval spores centrally in unswollen sporangia. Colonies are circular, rough, flat and erose, opaque, white and dry when grown on YEB plate. Catalase and urease are produced, oxidase and lipase are not produced. Voges-Proskauer test and Methyl Red reaction are positive. Nitrate is not reduced. Gelatin, starch and casein are hydrolyzed. There are no special growth factors requirements. Glucose can be used as a sole carbon source, but citrate cannot. Acids produced from glucose, trehalose, maltose and glycerol. No gas produced from glucose. Optimum growth temperature 30 °C and optimum pH 7.0. The G + C content of DNA is 39.8 mol %. The type strain is LQQ^T^.

## 3. Experimental Section

### 3.1. Bacterial Strain and Medium

The bacterium used for the production of autoinducer is the strain *P. carotovorum* isolated from Chinese cabbage leaves, which was grown on YEB plates at 30 °C. The indicator strain for determining the autoinducer activity is *Agrobacterium tumefaciens* NT1, which contains a *tra-lacZ* fusion and expresses β-galactosidase activity in the presence of a recognized autoinducer [20]. YEB agar medium contained (per 1,000 mL): 5 g sucrose, 5 g yeast extract, 0.5 g MgSO_4_·7H_2_O, 10 g tryptone, 5 g NaCl, 15 g agar, adjust pH to 7.0–7.2 with 0.1 M NaOH. The minimal medium constituted (per 1,000 mL) 10.5 g K_2_HPO_4_, 4.5 g KH_2_PO_4_, 0.2 g MgSO_4_· 7H_2_O, 5 mg FeSO_4_, 10 mg CaCl_2_, 2 mg MnCl_2_, 2 g (NH_4_) _2_SO_4_, 2 g d-Mannitol, 15 g agar, and pH 7.2. When necessary, a solution (5 mL) containing 1.5% (w/v) agar and 40 μg/mL 5-bromo-4-chloro-3-indoyl-β-d-galactopyranoside (X-gal) was overlaid onto the minimal medium plates. Basal medium used for the determination of taxonomic properties consists of (per 1,000 mL) 0.5 g MgSO_4_·7H_2_O, 0.7 g KNO_3_, 1 g, 0.5 g NH_4_Cl, 1 g NaCl (pH 7.0–7.2).

### 3.2. Isolation of Autoinducer-Digesting Strain

A soil sample (1 g) collected from Melbourne, Australia, was suspended in sterilized water (50 mL) and spread over the YEB agar plates in a tenfold serial dilution. After incubation at 30 °C overnight, some colonies were selected randomly and inoculated into YEB liquid medium. After incubation at 30°C with shaking for 36 h, 50 μL of the culture supernatant was mixed with the equal volume of 40 μM synthetic autoinducer (*N*-β-oxooctanoyl-l-homoserine lactone) and incubated at 30 °C for 3 h. A 5 μL mixed culture was removed for the detection of the target strain quenching AHL QS signal. One colony producing the maximum autoinducer quenching activity was chosen. To ensure the strain purity, the isolate was streaked on YEB medium plates and a single colony was used for further studies.

### 3.3. Bioassays for Detection of Autoinducer-Quenching Bacterium

Bioassays for determining the autoinducer-quenching strain were performed as described previously [21]. Briefly, minimal medium agar (25 mL) supplemented with 40 μg/mL of X-gal was cut into agar gel slices (in 0.5 cm width) every 0.5 cm on the plate. The mixed fluid (5 μL) of the culture supernatant and synthetic autoinducer being incubated at 30°C was spotted on the top of agar gel strips, and then the cell culture of *A. tumefaciens* NT1 (OD_600_ ≈ 0.4) was inoculated on the remaining agar gel strips from the loaded samples with an interval of 0.5 cm using a sterilized tip. The plates were incubated at 30 °C overnight. The distance from the last induced blue colony to the origin of the loaded sample in each agar slice was measured. No inoculum of the mixed fluid sample on the top of agar gel strip should be done to serve as a negative control. The blue colonies indicated the presence of autoinducer [22]. If the autoinducer was digested during incubation with the culture of the target strain, only white colonies or a few blue colonies will be observed on the plate after incubation, which suggested that the isolate has ability to quench autoinducer activity. The distance of blue colonies is in inverse proportion to the autoinducer-quenching activity. All bioassay experiments were performed in triplicate unless otherwise stated.

### 3.4. Morphological Characteristics

The unidentified culture was grown in the YEB medium at 30 °C for 16 h and observed with a phase-contrast microscope and transmission electron microscope. For transmission microscopy, bacterial cells were fixed with 5% (w/v) glutarldehyde and 1% (w/v) osmium tetroxide. Ultrathin sections of the sample embedded in epoxy resin were prepared with an ultramicrotome, stained with uranyl acetate and lead citrate, and examined with a model JEM-1200 EX transmission electron microscope (JEOL Ltd, Tokyo, Japan). Cellular morphology was also assessed after Gram staining and observed by light microscopy, and the morphology of the fixed specimens compared to that of living cells. The motility was observed in the hanging-drop mount as described previously [23]. Flagella staining were carried out with the Leifson staining method [23]. The AHL assay was performed as described elsewhere [21].

### 3.5. Physiological and Biochemical Properties

Gram staining characteristics were determined using the Hucker staining method as described by Murray *et al*. [23]. The oxidase test was performed by moistening a filter paper disk (7 cm in diameter) in a petri dish with 2 or 3 drops of 1% (w/v) tetramethyl-*p*-phenylenediamine dihydrochloride. A small amount of culture was smeared across the filter paper with a platinum loop, and the occurrence of a purple reaction within 30 s was considered as positive result. In the catalase test, 3% (w/v) hydrogen peroxide solution was pipeted onto a culture after incubation in YEB medium for 18–48 h and examined for the formation of gas bubbles. Urease activity was detected on Christensen urea agar slant [24] by presence of a red-violet color. Lecithinase and lipase activity was tested as described previously [24]. Tests for gelatin hydrolysis (method 1), indole production (method 2), hydrogen sulfide production (method 2), nitrate reduction, methyl red reaction, Voges-Proskauer reaction, citrate utilization (method 1) and hydrolysis of starch, casein and cellulose were carried out by using the methods previously described [24]. Acid and gas production from carbohydrates were determined in the basal medium supplemented with various carbohydrates as described [24]. The media used to evaluate utilization of various substrates for growth were prepared by adding 0.2% (w/v) of each substrate to a basal medium. In carbon source or nitrogen source utilization tests, the optical density at 600 nm of a culture after cultivation in each medium was compared with that in the basal medium.

The temperature range for cell growth was determined by inoculating a loopful of young culture onto YEB agar and incubating at the required temperature from 5 °C to 60 °C for 10 days and 2 days respectively. The pH ranges for cell growth were determined by incubating cells in YEB medium with different pH at 30 °C for 2 days.

### 3.6. DNA Base Composition and DNA-DNA Hybridization

DNA was extracted from cells grown at 30 °C overnight in YEB medium, and purified using the methods described by Moore *et al.* [25]. The G + C content of DNA was determined by the thermal denaturation method described by Marmur and Doty [26]. The DNA of *Escherichia coli* K-12 was used as a standard (G + C = 50.6 mol %). The values given are the means of the values from three separate experiments. Levels of DNA relatedness were determined by using non-radioactive detection system developed by Ziemke *et al.* [27].

### 3.7. 16S rRNA Gene Sequence Analysis

A 16S rRNA gene fragment of the species LQQ that corresponds to the position of 95 to 1395 of *Escherichia coli* 16S rRNA was amplified by PCR, using the purified DNA and a primer combination consisting of 5’-TGACGAGTGGCGGACGGGTG-3 (16sf95, forward primer) and 5’-CCATGGTGTGACGGGCGGTGTG-3’ (16sr394, reverse primer) as described elsewhere [28]. The following temperature cycles were performed: 94 °C for 3 min, 30 cycles of 94 °C for 30 s, 65 °C for 30 s and 72 °C for 100 s, followed by a final 7 min incubation at 72 °C. The amplification products were purified with a QIAquick PCR purification kit (Qiagen, Germany) and sequenced using dRhodamine terminator cycle sequencing kit (PE Applied Biosystems) and a model 2400 Perkin Elmer GeneAmp PCR System (PE Applied Biosystems) according to the manufacturer's protocol. Sequences were determined from both strands with a Perkin Elmer ABI PRISM 377 DNA sequencer. To avoid misreading due to PCR errors, sequencing of the PCR fragment was repeated at least twice.

### 3.8. Phylogenetic Analysis

The closely known relatives of the new LQQ isolate were determined by performing sequence searches in the GenBank/EMBL database using BLAST program [14] and at Ribosomal Database Project-II [15]. The 16S rRNA gene sequences of these closely related strains were retrieved from the databases. These sequences were aligned using CLUSTAL X program [29] and corrected manually. Only unambiguously aligned positions were used for phylogenetic analysis. Distance matrices were produced with the DNADIST program of the PHYLIP package [30] and a phylogenetic unrooted tree was constructed using the NEIGHBOR program contained in the PHYLIP software package (V3.6) [30]. The statistical signification of the groups obtained was assessed by bootstrapping (100 replicates) using the program SEQBOOT, DNADIST, NEIGHBOR and CONSENSE [30]. The percentage similarities of 16S rRNA gene sequence of strain LQQ with other closely relative bacteria were calculated using Megalin program in DNASTAR package (DNASTAR, Inc., Madison, WI, USA). The GenBank accession numbers are shown in Figure 3 and the similarity for strains examined in this paper was given in Table 2.

### 3.9. Bioassay for Potato Tuber Soft Rot

Inoculum for bioassaying severity of potato tuber soft-rot was prepared from 24-h-old cultures of individual strains grown at 30 °C on YEB plate by washing cells once in 10 mM phosphate buffer (pH 7.0) and suspending in the same buffer at cell density of 10^8^ CFU/mL. A 50 μL suspension of the isolate LQQ was mixed with 30 μL suspension of *P. carotovorum* prior to inoculation. Potatoes were cut into 1 cm-thick slices. The sliced potato tubers were surface sterilized with 0.5% sodium hypochlorite for 5 min and rinsed thoroughly with sterile distilled water. The sliced potatoes were transferred to sterile Petri dishes and 30 μL of the mixed suspension was inoculated on potato tuber. The potato tuber dishes were incubated at 25 °C for 48 h. The single-strain inoculation of *P. carotovorum* was necessary for positive test, which was prepared by mixed 30 μL suspension of *P. carotovorum* with 50 μL of 10 mM phosphate buffer (pH 7.0). Both single- and mixed-strain inoculation were applied on three sliced potato tubers, respectively.

## 4. Conclusions

A Gram-positive, facultatively anaerobic, endospore-forming and rod-shaped bacterium was isolated from soil. It is able to quench the AHL autoinducers produced by other bacteria leading to the disruption of QS system. In according to the phenotypic and phylogenetic analysis together with the data of DNA-DNA hybridization, the LQQ isolate should assigned to the genus *Bacillus* within a novel species, for which the name *Bacillus marcorestinctum* is proposed.

## Figures and Tables

**Table 1.** Differentiating characteristics of *Bacillus marcorestinctum.*

**Characteristic**	**LQQ^T^**	**Characteristic**	**LQQ^T^**
Straight Rod	+	Citrate utilization	−
Round-end	+	Hydrolysis of	
Diameter	0.7–1.0	Gelatine	+
Length	3.0–6.0	Starch	+
Occurrence		Cellulose	−
Single	+	Casein	+
Pair	−		
Chain	+	Glucose as carbon source	+
		Catabolism	Fermentation
Capsules and slime layer	−	H_2_S production from	
Gram	+	Cystein	+
Cyst and Microcyst	−	Triosulfate	−
Coccoid body	−	TSI	−
Endospore	+ (central)		
		M.R.	+
Motility	−	V.P.	+
Facultative anaerobic	+	Nitrate reduction	+
Catalase	+	Indole production	−
Oxidase	−	Gas production	−
Urease	+	Growth temperature (°C)	10–37
		Acid-fast test	+
Lecithinase	+	Acid production	+
Lipase	−		

Symbols: +, positive; −, negative.

**Table 2.** Similarity levels observed by comparison of 16S rRNA gene sequence of strain LQQ^T^ with the other closet representatives of the genus *Bacillus*.

**Species/accession number**	**Percent identity**	**Species/accession number**	**Percent identity**
*Bacillus acidiceler*	90.9	*Bacillus horikoshii*	89.6
DQ374637		X76443	
*Bacillus alkalitolerans*	94.6	*Bacillus luciferensis*	91.5
DQ148952		AJ419629	
*Bacillus anthracis*	96.5	*Bacillus megatrium*	90.0
DQ232746		D16273	
*Bacillus cereus*	96.4	*Bacillus mycoides*	96.2
AF290547		ACMU01000002	
*Bacillus cohnii*	89.6	*Bacillus panaciterrae*	90.9
X76437		AB245380	
*Bacillus flexus*	90.2	*Bacillus shackletonii*	90.4
AB021185		AJ250318	
*Bacillus funiculus*	90.6	*Bacillus thuringiensis*	96.5
AB049195		AF290545	
*Bacillus halmapalus*	90.7	*Brevibacillus brevis*	96.5
X76447		EU931557	

**Table 3.** Characteristics that distinguish *Bacillus marcorestinctum* LQQ^T^ from the other related *Bacillus* species.

**Characteristics**	**LQQ^T^**	**1**	**2**	**3**	**4**	**5**
Cells diameter > 1.0 μm	−	+	+	+	+	−
Anaerobic growth	−	+	+	+	+	−
Lipase	−		+	+	+	ND
Citrate utilization	−	d	+	d	+	d
Growth at 10 °C	+	−	d	d	d	−
Growth factor required	−		+	+	ND	+

Taxa: 1, *Bacillus anthracis*; 2, *Bacillus cereus*; 3, *Bacillus mycoides; 4, Bacillus thuringiensis*; 5, *Brevibacillus brevis*. Symbol: +, 90% or more of strains are positive; −, 90% or more of strains are negative; d, 11–89% of strains are positive; ND, no data available.
